# Psychiatric Comorbidities in Pediatric Inpatients With Human Immunodeficiency Virus Infection and Impact on Hospital Course: Inputs From a Case-Control Inpatient Study

**DOI:** 10.7759/cureus.15686

**Published:** 2021-06-16

**Authors:** Caroline C Dias, Victoria Ayala, Fartun A Aliduux, Sayeda A Basith, Albulena Sejdiu, Miles M Nakaska, Sabiha Akter, Keerthika Mathialagan, Pradipta Majumder

**Affiliations:** 1 Psychiatry, Yenepoya Medical College and Hospital, Mangalore, IND; 2 Psychiatry, Ross University School of Medicine, Bridgetown, BRB; 3 Medicine, Medical University of the Americas, Charlestown, KNA; 4 Psychiatry and Behavioral Sciences, Medical University of the Americas, Charlestown, KNA; 5 Psychiatry, Saints Cyril and Methodius University, Skopje, MKD; 6 Family Medicine, American University of the Caribbean, Calgary, CAN; 7 Psychiatry, Bergen New Bridge Medical Center, Paramus, USA; 8 Psychiatry, Sree Balaji Medical College and Hospital, Chennai, IND; 9 Psychiatry, Drexel University College of Medicine, Philadelphia, USA; 10 Psychiatry, WellSpan Health, York, USA

**Keywords:** adolescent health, human immunodeficiency virus infection, psychiatric comorbidities, depression, psychosis

## Abstract

Objectives

In this study, we aimed to delineate psychiatric comorbidities in pediatric inpatients with versus without human immunodeficiency virus (HIV) infection and to measure its impact on the length of stay (LOS) and cost of treatment during hospitalization.

Methodology

We conducted a case-control study using the Nationwide Inpatient Sample and included 4,920 pediatric inpatients between the ages of six and 18 years who were sub-grouped by a comorbid diagnosis of HIV (N = 2,595) and non-HIV (N = 2,325) and matched for demographics (age, sex, and race) by propensity case-control matching. Logistic regression analyses were used to evaluate the adjusted odds ratio (aOR) of association for psychiatric comorbidities (depression, anxiety, post-traumatic stress disorder, psychosis, and drug abuse) in the HIV-positive compared with the HIV-negative (as reference category) pediatric inpatients. We measured the differences in the LOS and cost using the independent sample t-test.

Results

We found that the most prevalent psychiatric comorbidities in the HIV-positive group were anxiety (6.9%), drug abuse (6.6%), psychosis (6.4%), and depression (6.2%). The HIV-positive group had a significantly higher likelihood of comorbid psychosis (aOR: 1.82; 95% confidence interval [CI]: 1.38-2.40) and depression (aOR: 1.79; 95% CI: 1.36-2.36). The mean LOS per hospitalization episode was longer for the HIV-positive group (11.1 days vs. 6.0 days; P < 0.001) compared to the HIV-negative pediatric inpatients.

Conclusions

We found an increased risk of depression by 79% and psychosis by 82% in the HIV-positive pediatric population. These inpatients also had an extended hospitalization stay (by five days), adding to the healthcare economic burden.

## Introduction

Human immunodeficiency virus (HIV) infection is a global epidemic that has affected around 38 million people worldwide, of which 2.8 million are children under 19 years of age [1]. In the United States, 645 children under 13 years of age were diagnosed with HIV infection from 2014 to 2018, an overall decline of 54% [2,3]. While children under 13 years of age acquired HIV infection perinatally, adolescents acquired the infection through sexual transmission [4]. The highest rate of diagnoses in children was among blacks, followed by Hispanics and whites in the United States [3]. Globally, around 110,000 children and adolescents died from acquired immunodeficiency syndrome (AIDS)-related causes in 2019, and about 70% of these deaths occurred among children less than 10 years of age [1].

While early childhood survival for children living with HIV has improved, less progress has been observed in adolescence [1]. Adolescents suffer from additional barriers in accessing testing and treatment as well as limited psychosocial support. Although access and quality of treatment have improved the lifespan of affected individuals, the quality of life in children is meager [5].

A systematic review investigating mental health issues found that the most common psychiatric comorbidities in the HIV-positive pediatric population were attention deficit hyperactivity disorder (28.6%), anxiety disorders (24.3%), and depression (25%) [6]. Other psychiatric comorbidities included conduct disorder and oppositional defiant disorder, with a prevalence rate of 13% and 11%, respectively [7]. There is an underlying pathophysiology between psychiatric conditions and HIV due to similar neurocircuitry and receptor systems [8]. Beyond this link, the neurotoxic effects of HIV infection and aberrant immune activation could potentiate a myriad of mental health issues. Psychosocial factors such as poverty, family history of substance use, psychiatric comorbidities, and violence could indirectly contribute to mental health issues in HIV-positive children and adolescents [9].

Despite the multitude of studies on HIV pathogenesis, comorbidities, and treatment in adults, this consideration has not been extended to the pediatric population. Past literature exploring psychiatric comorbidities in HIV-positive pediatric inpatients was limited by small sample sizes without comparison groups [10]. To address the limitations of previous studies, we included a larger sample and a control group matched for confounding demographic characteristics. We conducted a case-control inpatient-based study to evaluate the risk of psychiatric comorbidities in HIV-positive pediatric inpatients and measure the impact of psychiatric comorbidities on the length of stay (LOS) and cost of hospitalization.

## Materials and methods

Study sample

We conducted a case-control study using the Nationwide Inpatient Sample (NIS, 2012-2014). NIS is the largest inpatient database and represents a sample of 4,411 non-federal community hospitals across 44 states in the United States [11]. We included 4,920 pediatric inpatients hospitalized for medical conditions (age six to 18 years) who were sub-grouped by a comorbid diagnosis of HIV (N = 2,595) and non-HIV (N = 2,325) and matched for demographics (age, sex, and race) by propensity case-control matching and a match tolerance set at zero for exact matches using Statistical Package for the Social Sciences (SPSS) version 26.0 (IBM Corp., Armonk, NY).

Variables

We defined psychiatric comorbidities as any coexisting diagnoses in the inpatient record, and we included depression, anxiety, post-traumatic stress disorder (PTSD), psychosis, and drug abuse. We calculated the LOS as the number of nights the patient spent in the hospital for a primary diagnosis of psychiatric illness. Total cost during hospitalization excluded professional fees and non-covered charges [12].

Statistical analysis

We investigated the rates of psychiatric comorbidities in HIV-positive pediatric inpatients and compared them with HIV-negative group by performing descriptive statistics and Pearson’s chi-square test. Next, we measured the differences in continuous variables, i.e., LOS and cost in HIV-positive pediatric inpatients versus HIV-negative group using the independent sample t-test. A binomial logistic regression model was used to evaluate the adjusted odds ratio (aOR) of association for psychiatric comorbidities in the HIV group with the non-HIV group as the reference category. All analyses were conducted using SPSS version 26.0 (IBM Corp., Armonk, NY), and statistical significance was set to a two-sided P-value of < 0.05.

Ethical approval

The NIS is a publicly available de-identified data with the protection of patients, physicians, and hospital-related information; hence, we were not required to take approval from the institutional review board for our study [11].

## Results

HIV-positive pediatric inpatients were majorly females (55.5%) and blacks (61.5%). The demographic characteristics of the study participants after propensity case-control matching are shown in Table 1.

**Table 1:** Demographic characteristics of the propensity-matched study sample. SD: standard deviation

Demographic	HIV-negative	HIV-positive	P-value
N	2,325	2,595	-
Mean age (SD), in years	14.7 (3.8)	14.7 (15.9)	0.545
Sex, in %			
Male	42.8	44.5	0.227
Female	57.2	55.5	
Race, in %			
White	17.4	17.4	1.000
Black	61.5	61.5	
Hispanic	13.3	13.3	
Others	7.7	7.7	

The most prevalent psychiatric comorbidities in the HIV-positive group were anxiety (6.9%), drug abuse (6.6%), psychosis (6.4%), and depression (6.2%). Compared with the HIV-negative group, the inpatients in the HIV-positive group had a significantly higher likelihood of comorbid psychosis (aOR: 1.82; 95% confidence interval [CI]: 1.38-2.40) and depression (aOR: 1.79; 95% CI: 1.36-2.36). Although the prevalence of anxiety and drug abuse was higher, it had a statistically non-significant association, as shown in Table 2.

**Table 2:** Prevalence and odds of association of psychiatric comorbidities. PTSD: post-traumatic stress disorder

Comorbidity	Pearson’s chi-square test				Logistic regression analysis		
	HIV-negative, %	HIV-positive, %	Total, %	P-value	Adjusted odds ratio	95% Confidence interval	P-value
Depression	3.4	6.2	4.9	<0.001	1.79	1.36–2.36	<0.001
Anxiety	6.0	6.9	6.5	0.194	1.08	0.83–1.42	0.568
PTSD	1.7	1.9	1.8	0.590	0.89	0.54–1.47	0.652
Psychosis	3.4	6.4	5.0	<0.001	1.82	1.38–2.40	<0.001
Drug abuse	4.7	6.6	5.7	0.006	1.18	0.90–1.54	0.224

The mean cost per hospitalization in the HIV-positive group was $53,663, and there was statistically no significant difference compared with the HIV-negative group ($53,250; P = 0.907). However, the mean LOS per hospitalization episode was longer for the HIV-positive group (11.1 days vs. 6.0 days; P < 0.001), thereby increasing the mean LOS by five days, as shown in Table 3.

**Table 3:** Impact of psychiatric comorbidities on hospitalization course LOS: length of stay; SD: standard deviation; $: US dollars

Variable	Psychiatric comorbidity		Mean difference	P-value
	No	Yes		
Mean LOS (SD), days	6.0 (9.24)	11.1 (33.49)	5.1	<0.001
Mean cost (SD), $	53,250 (118,932)	53,663 (85,794)	413.4	0.907

## Discussion

Our study found higher rates of pediatric inpatients with HIV-positive in blacks, which coincides with the higher prevalence of HIV in this community [3]. A previous study found that a low socioeconomic status, disparities in healthcare access, HIV stigma, lack of awareness, and mistrust of the healthcare system could account for much higher rates of HIV infection among blacks [13].

The prevalence of depression and psychosis were significantly higher among HIV-positive pediatric inpatients, which are supported by previous studies and possibly attributed to a symbiotic relationship between HIV and the chronic inflammatory response [6,14]. Although we also found a higher rate of psychiatric comorbidities among HIV-positive pediatric inpatients, our rates were lower than those reported in previous studies [15]. Our findings are close to a cross-sectional descriptive study carried out in a pediatric clinic in Nairobi which included 162 HIV-infected children and adolescents (six to 18 years of age). The most common psychiatric diagnoses in this study were major depression (17.8%), social phobia (12.8%), and oppositional defiant disorder (12.1%) [16]. Meanwhile, a study from north Nigeria found a relatively low prevalence of psychiatric comorbidities including depression and psychosis [17].

HIV Infection leads to increased levels of pro-inflammatory cytokines and an imbalance in the immune response, which in turn contributes to the development of depression and depressive-like behaviors [18]. In addition, depression can harm the immune system by decreasing its cluster of differentiation 4 (CD4) cells, perpetuating a cycle [19]. Psychosocial factors such as stigma, fear of disease progression, and the burden of treatment cost could also potentiate the risk of depression in the HIV-positive group.

We also found an increased prevalence of psychosis in the HIV-positive group. A retrospective study conducted in the United Kingdom found an increased rate of psychosis among patients who acquired HIV perinatally [20]. The increased prevalence of psychosis could be due to perinatally acquired HIV infection and subsequent central nervous system involvement, lifetime exposure to the virus, and immune dysfunction during brain development among various other possible causes. In addition, treatment with anti-retroviral treatment like efavirenz alone or in combination with psychotropic medications may also precipitate psychosis [21].

Although the prevalence of anxiety and drug abuse was higher in the HIV-positive group, it had a statistically non-significant association. There have been limited studies exploring the association between anxiety among HIV patients. A cross-sectional study with a sample size of 195 children conducted in rural China found an increased association of anxiety among HIV-infected children. The increased association could be because the study measured five anxiety disorders: panic/somatic disorder, generalized anxiety disorder, separation anxiety disorder, social anxiety disorder, and school avoidance. Moreover, the study did not control the analytical model for confounding psychiatric comorbidities [22].

In addition, we studied the impact of psychiatric comorbidities on the hospitalization course. The difference in the mean cost per hospitalization in the HIV-positive group was statistically non-significant when compared with the HIV-negative group. Previous studies exploring care costs found that costs for HIV-related hospitalization increased only in HIV-positive groups with a very low CD4+ count. Hospitalization cost did not differ significantly between HIV-positive patients with high CD4+ and non-HIV-related hospitalizations [23]. However, we found the mean LOS per hospitalization episode was longer for the HIV-positive group, and this difference was statistically significant. In the pediatric population, medical conditions with comorbid psychiatric diagnoses could have led to increased hospitalizations, LOS, and overall cost of the hospital stay [14,24].

There were few limitations to our study. As a cross-sectional study, one of our limitations was that we could not find a causal relationship. In addition, because we used an administrative database, there was a lack of patient-level clinical information. Another limitation was that the diagnoses were based on the diagnostic codes, so there is a possibility of under-reporting of psychiatric comorbidities as compared to the existing literature we found a lower prevalence of anxiety and substance use in our study. The strength of our study is that we used NIS which enabled us to get a nationally representative sample of inpatients. Additionally, information is coded independently by individual practitioners, so there is minimal reporting bias.

## Conclusions

The HIV-positive pediatric inpatients had an increased risk of psychiatric comorbidities for depression (increased by 79%) and psychosis (increased by 82%) compared to the HIV-negative inpatients. A comorbid diagnosis of HIV in this at-risk pediatric population extended the hospitalization stay by five days, though it had no significant impact on the total hospitalization cost. Early diagnosis and management of psychiatric comorbidities among HIV-positive pediatric inpatients can improve the quality of life with better patient outcomes.
